# Observations in the Pathology and Treatment of Cholera

**Published:** 1874-08

**Authors:** 


*Observations in the Pathology and Treatment of Cholera. The Result of Forty Years' Experience.* By JOHN MURRAY, M. D. New York: G. P. Putnam's Sons, 1874.

Dr. Murray does not present any essentially new points in this little monograph, but gives in a brief way the results of his extensive experience with the disease in India. He gives no treatment for cholera which may be termed specific, but enjoins careful attention to each case, for he believes that a large number of cases may be saved by appropriate measures promptly administered. He embodies in this little book some excellent suggestions concerning cholera, and his discriptions of its various phases are very admirable. It presents to those who wish to look up the subject a convenient and valuable hand-book.

